# Endoscopic endonasal transsphenoidal surgery of 1,166 pituitary adenomas

**DOI:** 10.1007/s00464-014-3815-0

**Published:** 2014-10-01

**Authors:** Fuyu Wang, Tao Zhou, Shaobo Wei, Xianghui Meng, Jiashu Zhang, Yuanzheng Hou, Guochen Sun

**Affiliations:** Department of Neurosurgery, PLA 301 Hospital, Beijing, 100853 China

**Keywords:** Endoscopic surgery, Endonasal, transsphenoidal surgery, Pituitary adenoma

## Abstract

**Aim:**

To report the results of a series of patients undergoing pure endoscopic endonasal pituitary surgery and to evaluate the efficacy and safety of this procedure.

**Materials and methods:**

The data of 1,166 patients that underwent endoscopic endonasal transsphenoidal adenoma removal between December 2006 and June 2013 were retrospectively reviewed. Pre- and postoperative hormonal status (3 months after surgery) were analyzed and compared with the clinical parameters originally presented by the patients. The incidences of tumor removal, hormonal control, and tumor removal complications were retrospectively analyzed.

**Result:**

Out of 577 nonfunctioning adenomas, 180 were growth hormone (GH) secreting, 308 prolactin (PRL) secreting, 26 mixed GH/PRL adenomas, 68 adrenocorticotropin secreting, and 7 thyroid-stimulating hormone-secreting adenomas. The gross total removal of pituitary adenomas was achieved in 98 % of microadenomas, 92 % of macroadenomas, and 76 % of giant adenomas. Hormonal control was achieved in 47 (69 %) cases of ACTH adenomas, 119 (66 %) GH adenomas, 262 (85 %) PRL adenomas, and 6 (86 %) TSH adenomas. Postoperative complications were observed in 168 (14.4 %) patients. The most frequent complications were diabetes insipidus (7 %), epistaxis (1.7 %), hyposmia (1.5 %), anterior lobe insufficiency (1.3 %) ,and CSF leaks (0.6 %).

**Conclusion:**

The pure endoscopic approach is a safe, efficacious, and minimally invasive technique for the removal of pituitary adenomas. A higher gross total resection rate is vital for non-functional and functional adenomas. For patients with functional adenomas, while hormonal remission is unlikely to be achieved by surgery, the use of adjuvant therapy is advocated to obtain long-term hormonal control.

Pituitary tumors, one of the most frequent intracranial tumors, are clinically classified into 2 groups of tumors: functioning and nonfunctioning. Successful removal of the pituitary gland via the transsphenoidal approach was firstly successfully described by Schloffer [1] in 1906. The sublabial transsphenoidal approach to sellae turcica was then modified by Cushing in 1912 [2]. Following the introduction of the endoscope, innovative sellae, and parasellae surgery procedures are currently implemented. The endoscope-guided transsphenoidal surgery was standardized in clinical practice by Jho and Carrau [3] and Cappabianca et al. [4], introducing enhanced illumination and visualization of the lesions. In the current study, a review of a database of 1,166 consecutive pituitary adenomas, resected by conventional endoscopic endonasal transsphenoidal techniques was conducted. An analysis of the techniques, effectiveness and morbidity of the technique for pituitary adenomas by one doctor in a single center was conducted.

## Materials and methods

A total of 1,166 pituitary adenoma patients that underwent pure endoscopic endonasal transsphenoidal surgery, conducted by the leading surgeon Zhou tao at the Neurosurgery Department of PLA general hospital between January 2007 and June 2013, were retrospectively studied. All patients underwent neurologic, ophthalmologic, and endocrine examinations prior to surgery. The patient follow-up period varied from 3 months to 5.6 years.

## Endocrine assessment

All endocrine examinations were performed prior to and after surgery and at the time of follow-up consultation. Multiple measurements of plasma growth hormone (GH), insulin-like growth factor-I (IGF-I), prolactin (PRL), adrenocorticotrophic hormone (ACTH), cortisol, 24 h urinary free cortisol (when Cushing’s disease was suspected), thyroid-stimulating hormone (TSH), free thyroxine, luteinizing hormone (LH) and follicle stimulating hormone (FSH), testosterone, and estradiol (E2) levels were investigated.

## Neuroradiology

To evaluate the size and the invasion of the adenoma, all patients underwent magnetic resonance imaging (MRI), with and without administration of the intravenous contrast agent prior to surgery. Tumor size was classified in three categories according to maximum tumor diameter: microadenoma (<10 mm); macroadenomas (>10 mm); and giant adenoma (>40 mm). Conventional paranasal computerized tomography scans were used in 264 patients for surgical planning. Sphenoid sinus and septal anatomy were evaluated in detail. Follow-up MRI studies were obtained at 3 and 6 months following surgery.

## Surgical techniques

All patients were treated by the same medical team, using identical procedures. Under general anesthesia, the patient was placed in the supine position on the operative table with the back elevated at a 30° angle and the head tilted back at a 20° angle. The neurosurgeon was positioned on the right side of the patient and the assistant on the left side. Preoperative preparation of the nasal mucosa consists of pledgets soaked in tetracaine HCl 20 mg/ml and epinephrine HCl 0.0125 mg/ml mix solution which are placed on medial side of the turbinate through both nostrils for the mucosal decongestion and to decrease mucosal bleeding during nasal phase of surgery. Briefly, after intubation, the endoscope (Karl Storz, Tuttlingen, Germany) was inserted into the nasal cavity. The middle turbinate is lateralized or fractured to promote normal postoperative middle meatus physiology. In the reported cases, the space between the middle turbinate and the nasal septum was gently widened. The unilateral nostril was commonly utilized for the majority of patients, while bilateral nostrils were utilized for patients with giant adenomas or when the tumors were located in the sphenoid sinus. Predominantly, in the cases reported in this study, the right nostril approach was chosen. If the right nostril was unsuitable due to septal deviation or adenoma extension to the right cavernous sinus or to the superior sellae region, the left nostril was utilized. The ostium was observed and widened to visualize entrance to the sphenoid sinus. Drilling of the base of the sphenoid and particularly removal of the sphenovomerine suture enabled exposure of the sellae floor and also significantly increased the maneuverability of surgical instruments, thus augmenting the efficacy and safety of endoscopic surgery by improving accessibility to the tumor and controlling any bleeding, similar to that achieved by microsurgery. Inside the sphenoid sinus, exposure of the sellae diaphragm floor from one carotid protuberance to the other and craniocaudally from the planum sphenoidale to the clivus was achieved. The point of attachment of the diaphragm sellae should not be compromised with dual incision, which can be identified by preoperative MRI T2 imaging. Intrasphenoidal septal variations and the anterior wall of the sellae are easily evaluated by both coronal and sagittal paranasal CT scans preoperatively. Intercarotid distance is measured in great care with MRI and CT scans and these measures are rechecked intraoperatively with the aid of designed 10 mm scaled-dissector by the leading author. In most cases, the neuronavigation was used to identify diaphragm sellae, the anterior wall of the sellae and the dura mater are opened with a highspeed drill or Kerrison rongeur. Visualization of the sellae region was initially performed with a 0° endoscope. If a macroadenoma was encountered directly beneath the dura, decompression was commenced immediately. The interior was debulked in the traditional fashion using curettes and suction. In the current study, if a cavity opened during surgery, the endoscope was inserted into the cavity, allowing direct visualization of the residual tumor. Following tumor resection, 45° angled endoscopes were inserted into the surgical cavity to explore for any residual tumor. In some cases, the intraoperative MRI scan was performed to detect the residual tumor for further resection in case of macro adenomas. For the reconstruction of the sellae region, a small piece of Surgicel (Ethicon Ltd., U.K.) was placed into the sellae cavity, and Duragen (Integra, Plainsboro, NJ, USA), positioned as an overlayer graft in the intradual space. A larger piece of Duraform was then placed into the sinus to sustain the Duragen graft and Bio Glue (CryoLife, Inc., Kennesaw, GA, USA), applied to fix the graft. The expanding sponge was inserted into the window of the anterior wall of the sphenoidal sinus as a buttress and removed 48 h after surgery. In some cases that postoperative CSF leakage was obvious, an external lumbar drainage system was applied and kept in place for 5 days after surgery. If the postoperative CSF leakage continues, a second dual fixation operation should be made, a combination of duraform, underlayer, and overlayer free fascia lata grafts was placed in the sellae floor. In the 24 h after the surgery, CT and endocrinological evaluations were conducted. Hormonal assessments, identical to the preoperative hormonal screenings, were performed on postoperative day 1 and 7 and in the first and third postoperative months.

## Results

A total of 1,166 pituitary adenomas were treated by one surgeon in one medical center. Males and females represented 44.3 and 55.7 % of patients, respectively. The mean age of the population was 40.3 ± 15.25 years (Table 1). According to the size of the lesion, 245 (21.0 %) of tumors were classified as microadenomas, 806 (69.1 %) tumors as macroadenomas, and 115 (10 %) tumors as giant adenomas. Hormone-secreting adenomas represented the majority of lesions (589 cases, 50.6 %). The most common of functioning lesions was PRL secreting pituitary adenomas (26.4 %), followed by GH-secreting pituitary adenomas (15.4 %), mixed GH/PRL secreting pituitary adenomas (2.2 %), and ACTH-secreting adenomas (5.8 %), followed by TSH secreting adenomas (0.6 %) (Table 2).

**Table 1:** Characteristics in 1,166 patients with pituitary adenomas

Characteristics	*N* = 1,166
Gender	
Male *n* (%)	517 (44.3)
Female *n* (%)	649 (55.7)
Age (years)	40.3 ± 15.25
Median follow-up period (years)	2.40 (0.25–5.60)

**Table 2:** General characteristics of 1,166 pituitary adenomas

Adenomas characteristics	*N* (%)
Nonfunctioning adenomas	577 (49.5)
Hormone-secreting adenomas	589 (50.5)
GH-secreting adenomas	180 (15.4)
Prolactinoma	308 (26.4)
Mixed GH/PRL adenomas	26 (2.2)
ACTH-secreting adenomas	68 (5.8)
TSH secreting adenoma	7 (0.6)
Tumor size	
Microadenomas ≤ 10 mm	245 (21.0)
Macroadenomas ≤ 40 mm	806 (69.1)
Giant adenomas > 40 mm	115 (9.9)

*N* sample size

Gross total resection as ascertained by MRI results 3 months after surgery was achieved in 240 (98 %) cases of microadenomas, 742 (92 %) cases of macroadenomas, and 87 (76 %) cases of giant adenomas (Table 3). Visual recovery was acquired in 105 (92 %) cases, with the III and VI cranial nerve function restored in 22 (88 %) patients. Hormonal control, determined in the 3 months after surgery, was achieved in 47 (69 %) cases of ACTH-secreting pituitary adenomas, 119 (66 %) cases of GH-secreting pituitary adenomas, 262 (85 %) cases of PRL secreting pituitary adenomas, and 6 (86 %) cases of TSH secreting adenomas (Table 4).

**Table 3:** Results of gross total resection of pituitary adenomas

	*N*	Gross total resection *n* (%)
Microadenomas	245	240 (98)
Macroadenomas	806	742 (92)
Giant adenomas	115	87 (76)

*N* sample size

**Table 4:** Results of hormonal control

Hormone	*N*	Hormonal control *n* (%)	Microadenoma hormonal control (%)	Macroadenoma hormonal control (%)
GH	180	119 (66)	79	60
PRL	308	262 (85)	95	77
ACTH	68	47 (69)	59	71
TSH	7	7 (100)	100	None

*N* sample size

## Complications

Complications were observed in 157 (13.5 %) patients (Table 5). Diabetes insipidus (DI) was observed in 82 patients following surgery, 74 of them (6.3 % of all patients) presented with only transient dysfunction, while 8 patients presented with permanent DI, requiring vasopressin therapy and clinical follow-up. Postoperative CSF leakage was observed in 7 cases (0.6 %). A second operation was performed in these patients to reconstruct the sellae and the double layer of duraform and free fascia lata grafts placed in both the intradural and epidural space supported by fat tissue. Lumbar drainage was maintained for 5–7 days after the operation. Twelve patients that suffered from meningitis were adequately treated, and recovered well. Twenty patients suffered from postoperative epistaxis, 17 of which required coagulation by packing of the nasal cavity. One case was treated by the ENT colleagues, while 2 cases that did not respond to the above-mentioned method were treated with endovascular embolization of maxillary artery branches. Out of seventeen patients that suffered from transient hyposmia, 8 patients recovered after 3 months and 9 patients with giant nonfunctioning adenoma recovered after 6 months. Fifteen patients experienced hypopituitarism and consequently required thyroid replacement therapy and/or steroid replacement therapy. One patient presented with headache and transient III cranial nerve palsy. The CT scan of this patient showed SAH and consequently CTA was performed to exclude the aneurysm. One giant non-functional patient suffered coma after surgery and was lost when follow-up. Eight cases of intrasellae bleeding following surgery were observed. Evacuation of the hematoma via an endoscopic approach was performed in 7 cases, and a third operation was performed in one patient with residual hematoma after second evacuation surgery. Normal visual acuity was restored in 3 out of these 8 patients while 5 patients had poor visual acuity.

**Table 5:** Surgery complications in the 1,166 patients with pituitary adenomas

Type of complication	*n* (%) of patients
Transient diabetes insipidus	74 (6.35)
Permanent diabetes insipidus	8 (0.69)
Postoperative hemorrhage	8 (0.69)
CSF leak	7 (0.60)
Meningitis	12 (1.03)
Decreased visual acuity	5 (0.43)
Epistaxis	20 (1.72)
Anterior lobe insufficiency	15 (1.29)
SAH	1 (0.09)
Hyposmia	17 (1.46)
Coma	1 (0.09)
Total	168 (14.41)

## Case illustration

**Fig. 1:**
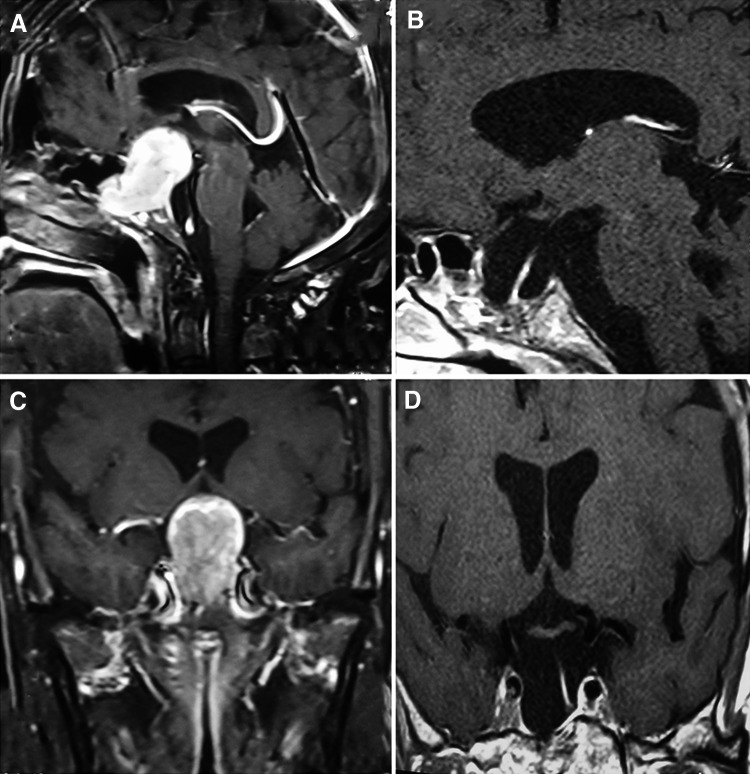
MRI images of one nonfunctioning adenoma. **A**, **C** Before surgery, **B**, **D** gross total resection after surgery

**Fig. 2:**
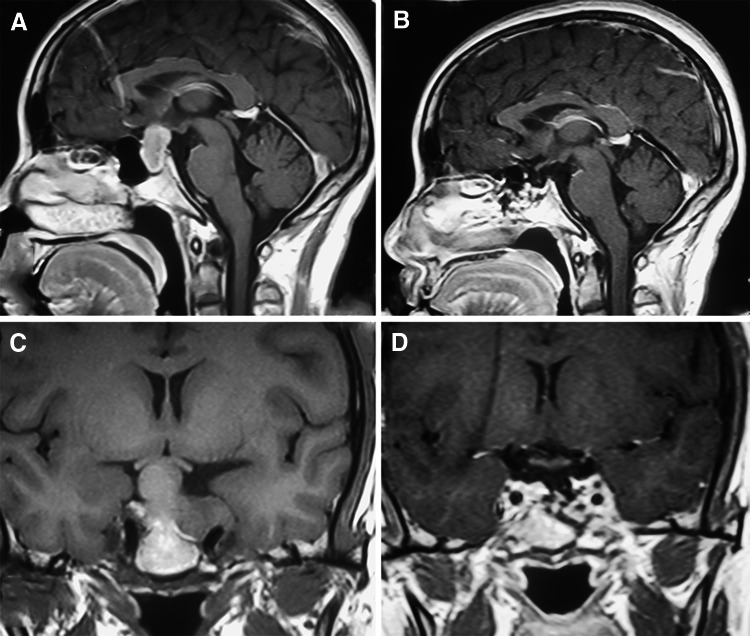
MRI images of one nonfunctioning adenoma invading cavernous sinus. **A**, **C** Before surgery, **B**, **D** gross total resection after surgery

**Fig. 3:**
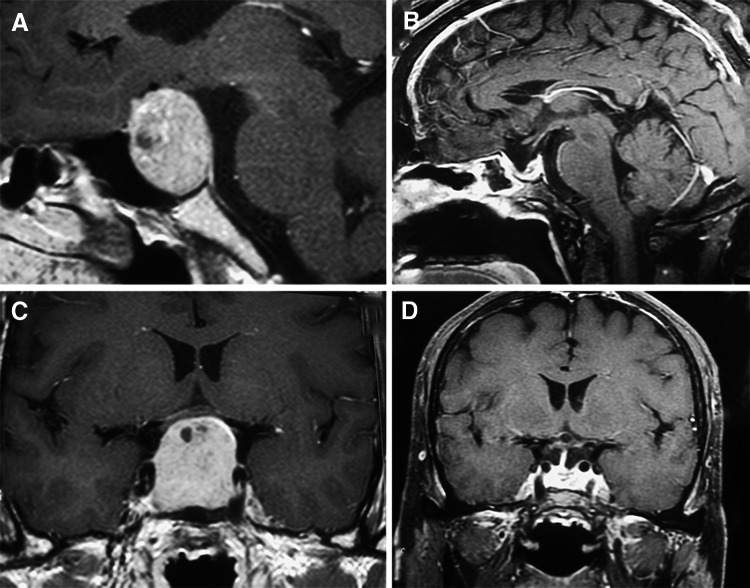
MRI images of one PRL adenoma. **A**, **C** Before surgery, **B**, **D** gross total resection after surgery

**Fig. 4:**
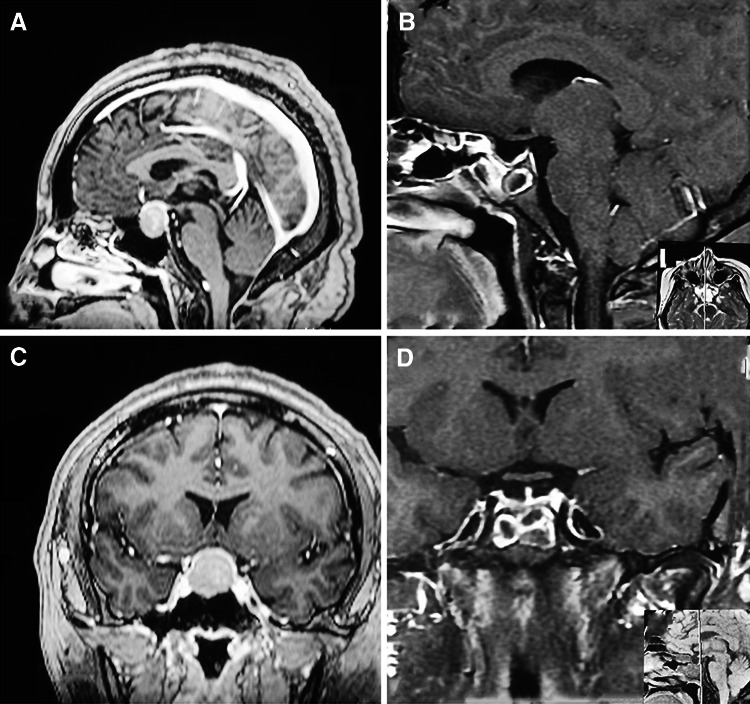
MRI images of one ACTH adenoma. **A**, **C** Before surgery, **B**, **D** gross total resection after surgery

**Fig. 5:**
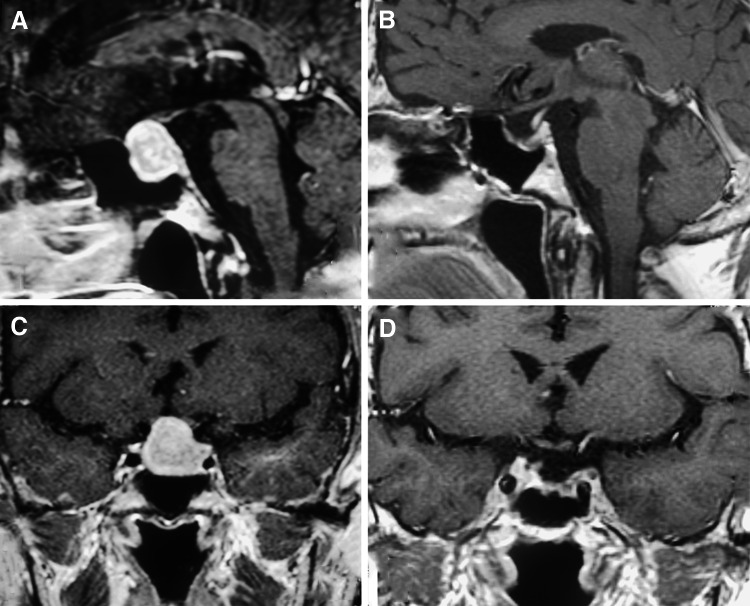
MRI images of one TSH adenoma. **A**, **C** Before surgery, **B**, **D** gross total resection after surgery

Five cases were listed (Table 6): 1 nonfunctioning adenoma (Fig. 1), 1 nonfunctioning adenoma invading cavernous sinus (Fig. 2), 1 PRL adenoma (Fig. 3), 1 ACTH adenoma (Fig. 4), and 1 TSH adenoma (Fig. 5). All the adenomas got gross total resection.

**Table 6:** Case illustration

	Gender	Age (years)	Type	Image before and after surgery	Resection	Hormonal control
1	F	66	Nonfunctioning	Figure 1	Gross total	–
2	F	35	Nonfunctioning	Figure 2	Gross total	–
3	M	45	PRL	Figure 3	Gross total	Yes
4	F	27	ACTH	Figure 4	Gross total	Yes
5	M	60	TSH	Figure 5	Gross total	Yes

## Discussion

In recent years, endoscopic endonasal transsphenoidal surgery has a wide application in the treatment of pituitary adenomas. It has been used in our medical center where the correspondence author (Doctor Zhou) of this manuscript is since 2007 because the endoscopic surgery can provide better vision and result in less nasal cavity injuries than the microsurgical approach.

Exclusive endoscopic transsphenoidal resection of pituitary adenomas is safe and efficacious when compared to the traditional microscopic approach. The endoscopic transsphenoidal approach provides a panoramic vision inside the surgical area, a superior close up of the anatomy, an improved working angle and less nasal cavity injuries. D’Haens et al. [5] reported the endocrinologic outcome in 2 series of patients using these 2 different techniques, the overall remission rate of hypersecretion was 63 % in the endoscopic group compared to 50 % in the microsurgical group; in noninvasive macroadenomas, the remission rate of hypersecretion obtained using endoscopy was 78 % compared with 43 % using microsurgery; the endocrinological results achieved for microadenomas were similar in the 2 groups; postoperative CSF leaks occurred more frequently in the endoscopic group (6 cases). Strychowsky et al. [6] made a systematic review included one prospective and nine retrospective articles, and demonstrated that the pure endoscopic approach was associated with less mean blood loss, shorter hospital stays and operative times, and fewer nasal complications, and a trend toward better gross total resection and decreased incidence of postoperative DI.

The removal rate of pituitary adenomas is closely related with tumor volume [7, 8], which was a predicting factor for the surgical outcome. Compared with traditional open or transsphenoidal microscopic methods, the endoscopic endonasal transsphenoidal approach offers the potential for aggressive resection via a minimal access corridor. In the current study, the majority of tumors were macroadenomas (69.1 %) and giant adenomas (10 %), and the gross total of resections of macroadenomas was 92 %, which was comparable to the results (96 %) reported by Dehdashti et al. [9]; the gross total of resections of giant adenomas was 76 %, which was higher than the results (47.2 %) of a systematic review conducted by Komotar et al. [10]. The higher gross total resection in the present study may be attributed to the use of intraoperative MRI and neuronavigation during the surgery. In our medical center, the intraoperative MRI and neuronavigation are often used during the treatment of macroadenomas bigger than 3 cm. After the initial resection, the intraoperative MRI scans can be used for assessing if there is residual tumors and determining if a second or third surgery is required for safe resection of the lesion. The surgery should not be continued if the tumor residue is out of reach, the access corridor is very narrow for the operation, or when most parts of some giant adenoma were removed to achieve the decompression. A second transcranial surgery or radiotherapy or radiosurgery may be considered for tumors invading into cavernous sinus and neighboring cranial base that cannot be totally removed by the first surgery.

### Nonfunctioning adenomas

The main symptoms of nonfunctioning adenomas include pituitary failure (hypopituitarism) and hemianopsia, progressive loss of vision, diplopia, headaches, and so on. Generally, nonfunctioning microadenomas are not suggested for surgical treatment. But for young patients with asymptomatic macroadenomas, the resection is recommended, if the suggestion is not accepted by the patient, close follow-up is necessary. When nonfunctioning macroadenomas produce acute intratumoral hemorrhage, resulting in symptoms such as sudden headaches, progressive loss of vision and diplopia, the immediate decompression treatment should be performed. Giant nonfunctioning adenomas may extend around the carotid artery, through the cavernous sinus, and into the temporal lobe or suprasellarly into the third ventricle and basal cisterns. In such cases, the transcranial surgery should be considered. If the surgery is performed by endoscopic approach, care must be taken not to damage the carotid artery while dealing with the carotid artery, and not to injury the vital structures such as the optic apparatus while dealing with the suprasellae part of the tumor. If the nonfunctioning adenoma could not be resected totally and are stable with no obvious symptoms in follow-up visit, it can be for further observation [11]. If it is developed, a second endonasal surgery or craniotomy may be considered.

### Functioning adenomas

Both PRL and GH secreting adenomas are the most common functioning adenomas. The data from recent literatures (Table 7) showed that the hormone control was higher for PRL secreting adenomas than that for GH secreting adenomas, and higher in microadenoma than macroadenoma. Our results revealed the similar trend (Table 4). Previous studies demonstrated that medical therapy has good effect in the treatment of PRL secreting adenomas, the patients with drug treatment had normal menstrual cycle and got pregnant, and thus medical therapy can be used as the preferred treatment for PRL secreting adenomas. Surgery is recommended when patients are intolerant to side effect of drugs, or cannot be cured by drugs, or suffer from pituitary apoplexy. However, in China, costs for medication are often considered, when patients select methods for the treatment of PRL secreting adenomas. After weighing the risk and benefit of surgery, many patients may select surgical treatment for PRL secreting adenomas in China. Preoperative medical therapy can reduce the tumor size and facilitate surgery for the giant PRL secreting adenomas.

**Table 7:** Hormonal control of functional pituitary adenomas resection from major endoscopic pituitary series in literature in the last 5 years

	Year	*n*	Type	Microadenoma hormonal control (%)	Macroadenoma hormonal control (%)
Hofstetter et al. [15]	2011	86	GH	75	40
			PRL	92.3	57.1
			ACTH	54.5	71.4
Gondim et al. [29]	2010	135	GH	81.8	68
			PRL	100	82.3
			ACTH	75	62.5
			FSH-LH	71.4	
			TSH	100	
Campbell et al. [13]	2009	26	GH	75	54.5
Yano et al. [30]	2009	29	GH	87.5	65.2
			PRL	94.1	41.7
Macchia et al. [16]	2009	26	TSH	67	40
Choe et al. [14]	2008	12	GH	100	87.5
			ACTH	100	

Surgery is preferred for the treatment of GH macroadenomas. The normalization of hormone levels is import in reducing the morbidity associated with tumors during the treatment. It should be noted that the physical condition of patients with poor cardiac function and physical condition should be improved by drugs such as somatostatin prior to surgery. In order to control hormone levels, tumors should be removed as much as possible so as to decrease the size of the adenoma for the followed medical therapy and radiotherapy. Tumor residue is a main cause of poor control of hormone level after surgery, cavernous sinus invasion was associated with a significantly lower remission rate in GH-secreting macroadenomas [12–15]. Ceylan et al. [12] demonstrated that endoscopic transsphenoidal surgery offered a wide exposure for cavernous sinus medial wall adenomas, enabling more removal of the adenoma from the medial cavernous sinus wall.

During the surgery for ACTH-secreting adenomas, the poor control of hormone level is often caused by poor visualization of lesions on preoperative MR imaging [15]. In our medical center, all the ACTH-secreting adenomas are surgically treated. The surgery is required to incise the adenomas while retaining the normal parenchyma of the gland as much as possible. In cases of the exact lesion site of ACTH-secreting adenomas invisible on preoperative MR imaging, the surgical treatment should be based on analysis of blood samples from preoperative inferior petrosal sinus.

Macchia et al. [16] reported that 55 % of TSH adenoma patients obtained remission (success rate of 40 and 67 % in macro- and microadenomas, respectively) following surgery. Pre-treatment with somatostatin analogs (SSA) resulted in an apparent, although not statistically significant increase in success rate in micro-, but not macroadenomas. A varied combination of surgery, somatostatin medicine, and radiotherapy can achieve long-term therapeutic effect [17, 18].

### Complications

Complications of the endoscopic surgery have an incidence of 3.4–36.1 % [7, 19–27], and are mostly DI, anterior lobe dysfunction, and CSF leak (Table 8). Temporary DI is thought to be a result of temporary dysfunction caused by surgical trauma, and stretching of pituitary stalk by the falling diaphragm after the removal of the adenoma. In the current study, all the patients with DI were cured after treatment. The anterior temporary lobe dysfunction might be related with operation of the pituitary gland such as the excessive use of the aspirator in the sellae cavity, inappropriate manipulation and resection of the normal pituitary gland neighboring the adenoma tissue, and heat damage of bipolar coagulation in sellae cavity to pituitary gland. Thus, hemostatic Surgicel rather than bipolar coagulator should be used for hemostasis in vicinity of the stalk [19]. In the macro- or giant adenoma, the pituitary tissue thinned by compression was difficult to distinguish from tumor and easy to be damaged when removing tumor, or the normal pituitary lie below the tumor and was also injured easily. Therefore, the relation between the tumor and normal gland should be carefully identified from the preoperative MRI image, in order to preserve the pituitary tissue during the surgery. The scraper ring was suggested to be used for adenoma resection because it could decrease the injury to normal tissue and diaphragm. The angled endoscope could be used, since it could facilitate the detection of the residual tumor and avoid damage of pituitary gland during the operation.

**Table 8:** Complications from endoscopic pituitary series in literature in recent 5 years

	Year	*N*	Total complication (%)	Most frequent complication (%)
Berker et al. [19]	2012	570	11.3	Diabetes insipidus (4.6)
Cavallo et al. [20]	2012	59	3.4	CSF leak (1.7)
				Hematoma in the tumor field (1.7)
Gondim et al. [21]	2011	301	26.9	Anterior lobe insufficiency (11.6)
De Witte et al. [22]	2011	83	36.1	Anterior lobe insufficiency (19.2)
Zada et al. [23]	2010	169	10.7	Epistaxis (3)
				Diabetes insipidus (3)
Gondim et al. [29]	2010	228	13.9	CSF leak (3.1)
Charalampaki et al. [24]	2009	150	19.7	Diabetes insipidus (5.9)
Yano et al. [30]	2009	213	10.8	CSF leak (4.2)
Dehdashti et al. [7]	2008	200	9.5	CSF leak (3)
				Anterior lobe insufficiency (3)
Present study		1,166	14.4	Diabetes insipidus (7)

Attention should be paid to intraoperative CSF leakage. Intraoperative CSF leakage could be found during or after the tumor removal. It could occur during the exploration of cavernous sinus and diaphragmatic recesses, and most of them could stop without any treatment. CSF leakage frequency and persistence increased in patients who had previous surgery, or in patients with suprasellae extension of the macroadenoma and during extended endoscopic approaches. CSF leak risk is higher in macroadenomas than microadenomas [25, 28], primarily due to the fact that the surgeon works closer to the diaphragm sellae and the subarachnoid space in these cases. Hence, it is critical to identify the tear in the diaphragm and/or arachnoid membrane during the surgery and seal it immediately [19]. In this study, the incidence of CSF was not high probably because (1) based on the point of attachment of diaphragma sellae identified on MRI T2 images, the sellae was open to a moderate extent in the anterior cranial base, avoiding laceration of the dura mater. (2) the diaphragm was kept intact during surgery. Macro- and giant adenomas often extended to the diaphragm. During the surgery, the diaphragm sella may fall to cover the remained tumors after parts of tumor were incised. In this case, further tumor resection could often damage the diaphragm sella. To keep the diaphragm intact, we identified the location of residual tumor and its relationship with the diaphragm sella using the intraoperative MRI scan, rescheduled the navigation. If the residual tumor located above or behind the falling diaphragm, the diaphragma was pushed upward in order to resect the residual adenoma, thus preventing breakage of the diaphragm. Rather, the surgeons chose opted removal of the tumor by inserting the endoscope to the tumor cavity. (3) CSF leak was repaired intraoperatively as soon as it was detected, in these cases, a strict reconstruction with double layer duraform is performed. Not all the CSF leak was from the broken diaphragm. In our study, 7 cases of CSF leakage after first surgery were reported. In the secondary operation, it was found that the CSF leak was from the laceration point of the dual of dorsum sellae in 2 cases, and damaged dual dorsum sellae by the infiltrating of the tumor in 1 case. All of these 3 cases showed no CSF leak in the first surgery. In the remaining 4 cases, the leakage lied in the diaphragm, with 3 cases showing no CSF leak in the first operation, and 1 case receiving repair when leak occurred, but with the duraform found to be leaking into the cavity following failed fixation. All the 7 cases had good outcome following the second repair.

The syndrome of inappropriate ADH (SIADH) was low and temporary (data not shown). Hyponatremia often occurred 5–10 days after surgery. Patients were treated with water restriction plus hypertonic sodium replacement.

## Conclusion

The exclusive endoscopic approach is a safe, efficacious, and minimally invasive technique for treatment of pituitary adenoma. A higher gross total resection rate is vital for non-functional and functional adenomas. For functional adenomas, hormonal remission is unlikely to be achieved by surgery alone, indicating that the use of adjuvant therapy be introduced to obtain long-term hormonal control. The progression of endoscopy instruments, more experienced doctors, and the development of more effective drugs are all important factors for the good outcome of pituitary adenoma.
